# The Efficacy and Safety of Eravacycline in the Treatment of Complicated Intra-Abdominal Infections: A Systemic Review and Meta-Analysis of Randomized Controlled Trials

**DOI:** 10.3390/jcm8060866

**Published:** 2019-06-17

**Authors:** Shao-Huan Lan, Shen-Peng Chang, Chih-Cheng Lai, Li-Chin Lu, Chien-Ming Chao

**Affiliations:** 1 School of Pharmaceutical Sciences and Medical Technology, Putian University, Putian 351100, Fujian, China; shawnlan0713@gmail.com; 2 Department of Pharmacy, Chi Mei Medical Center, Liouying 73657, Taiwan; httremoon@ms.szmc.edu.tw; 3 Department of Intensive Care Medicine, Chi Mei Medical Center, Liouying 73657, Taiwan; dtmed141@gmail.com; 4 School of Management, Putian University, Putian 351100, China; jane90467@gmail.com

**Keywords:** eravacycline, complicated intra-abdominal infection, efficacy, safety, mortality

## Abstract

This study aims to assess the clinical efficacy and safety of eravacycline for treating complicated intra-abdominal infection (cIAI) in adult patients. The PubMed, Web of Science, EBSCO, Cochrane databases, Ovid Medline, Embase, and ClinicalTrials.gov were searched up to May 2019. Only randomized controlled trials (RCTs) that evaluated eravacycline and other comparators for the treatment of cIAI were included. The primary outcome was the clinical cure rate at the test-of-cure visit based on modified intent-to-treat population, microbiological intent-to-treat population, clinically evaluable population, and microbiological evaluable population, and the secondary outcomes were clinical failure rate and the risk of adverse event. Three RCTs were included. Overall, eravacycline had a clinical cure rate (88.7%, 559/630) at test-of-cure in modified intent-to-treat population similar to comparators (90.1%, 492/546) in the treatment of cIAIs (risk ratio (RR), 0.99; 95% confidence interval (CI), 0.95–1.03; *I*^2^ = 0%, Figure 3). In the microbiological intent-to-treat, clinically evaluable, and microbiological evaluable populations, no difference was found between eravacycline and comparators in terms of clinical cure rate at test-of-cure (microbiological intent-to-treat population, RR, 0.99; 95% CI, 0.95–1.04; *I*^2^ = 0%, clinically evaluable population, RR, 1.00; 95% CI, 0.97–1.03; *I*^2^ = 0%, microbiological evaluable population, RR, 0.98; 95% CI, 0.95–1.02; *I*^2^ = 0%). In addition, eravacycline had clinical failure rate similar to comparators at test-of-cure in modified intent-to-treat population (RR, 1.01; 95% CI, 0.61–0.69; *I*^2^ = 0%), microbiological intent-to-treat population (RR, 1.34; 95% CI, 0.77–2.31; *I*^2^ = 16%), clinically evaluable population (RR, 1.03; 95% CI, 0.61–1.76; *I*^2^ = 0%), and microbiological evaluable population (RR, 1.32; 95% CI, 0.75–2.32; *I*^2^ = 10%). Although eravacycline was associated with higher risk of treatment-emergent adverse event than comparators (RR, 1.34; 95% CI, 1.13–1.58; *I*^2^ = 0%), no significant differences were found between eravacycline and comparators for the risk of serious adverse event (RR, 1.04; 95% CI, 0.65–1.65; *I*^2^ = 0%), discontinuation of study drug because of adverse event (RR, 0.68; 95% CI, 0.23–1.99; *I*^2^ = 13%), and all-cause mortality (RR, 1.09; 95% CI, 0.41–2.9; *I*^2^ = 28%). In conclusion, the clinical efficacy of eravacycline is as high as that of the comparator drugs in the treatment of cIAIs and this antibiotic is as well tolerated as the comparators.

## 1. Introduction

In contrast to uncomplicated abdominal infections, complicated intra-abdominal infections (cIAIs) can extend beyond the originally infected organ into peritoneal spaces, and can be associated with local or diffuse peritonitis [1,2]. *Enterobacteriaceae*, especially *Escherichia coli* and *Klebsiella pneumoniae*, are the most common pathogens causing cIAIs [3,4,5]. Emergence of multiple antibiotic resistances has become the major concern in this clinical entity and further limits the choice of optimal antibiotic treatment. *E. coli*, *Proteus* species, and *K. pneumoniae* are the most common pathogens; however, high resistance to broad-spectrum antibiotics, including extended-spectrum β-lactams and fluoroquinolones, among these pathogens, also emerges as a critical threat worldwide.

Eravacycline is a novel, synthetic fluorocycline antibacterial agent [6], and has excellent bactericidal activity against most antibiotic-resistant pathogens according to several in vitro studies [7,8,9,10]. Recently, the clinical efficacy of eravacycline in cIAI has been evaluated in several clinical studies [11,12,13]. However, an updated meta-analysis comparing the efficacy and safety of eravacycline and other comparators for the treatment of cIAI is lacking. Therefore, we conducted this meta-analysis to provide real-time evidence about the efficacy and safety of cIAI.

## 2. Methods

### 2.1. Study Search and Selection

All clinical studies were identified through a systematic review of the literature in PubMed, Web of Science, EBSCO, Cochrane databases, Ovid Medline, Embase, and ClinicalTrials.gov until May 2019 using the following search terms: “eravacycline”, “Xerava™”, “TP-434”, and “abdom*” (Search strategy presented in Appendix A). Studies were considered eligible for inclusion if they directly compared the clinical efficacy and safety of eravacycline with other antimicrobial agents in the treatment of adult patients with cIAIs. Studies were excluded if they focused on in vitro activity, animal studies, or pharmacokinetic–pharmacodynamic assessment. Two authors (S.-P.C. and S.-H.L.) searched and examined publications independently. When they disagreed, the third author (C.-C.L.) resolved the issue. The following data including year of publication, study design, type of infections, patients’ demographic features, antimicrobial regimens, clinical and microbiological outcomes, and adverse effects were extracted from every included study.

### 2.2. Outcome Measurement

The primary outcome of this meta-analysis was clinical response assessed at the test-of-cure visit, end-of-treatment, and follow-up visit based on modified intent-to-treat population, microbiological intent-to-treat population, clinically evaluable population, microbiological evaluable populations. The intent-to-treat population included all randomized patients, and the modified intent-to-treat population included all intent-to treat patients who received any amount of study drug. The microbiological intent-to-treat population included all modified intent-to-treat patients who met the minimal disease definition of cIAI and had a baseline pathogen identified. The clinically evaluable population included all modified intent-to-treat patients who met the minimal disease definition of cIAI and had a clinical response assessed at the test-of-cure visit. The microbiological evaluable population included all clinically evaluable patients who had a baseline pathogen identified and a microbiological response assessed. Clinical response was classified as cure, failure, or indeterminate based on clinical outcomes. Clinical cure was defined as resolution of all or most pretherapy signs or symptoms with no further requirement for antibiotics, radiological intervention, or surgery. The safety population included all patients who received any intravenous study therapy. Treatment-emergent adverse events were defined as adverse events that started during or after the first dose of study drug administration or increased in severity or relationship to the study drugs during the study. Serious adverse event is defined as an untoward medical occurrence or effect that at any dose results in death, is life-threatening, requires hospitalization or extension of existing hospitalization, or results in persistent or significant disability.

### 2.3. Data Analysis

The quality of enrolled RCTs and the risk of bias were assessed using Cochrane Risk of Bias Assessment tool [14]. Statistical analyses were conducted using the software Review Manager, version 5.3. The degree of heterogeneity was evaluated with the Q statistic generated from the χ^2^ test. The proportion of statistical heterogeneity was assessed using the *I*^2^ measure. Heterogeneity was considered significant when the *p* was less than 0.1 or *I*^2^ was greater than 50%. The random-effects model was used when data were significantly heterogeneous and the fixed-effect model was used when data were homogeneous. Pooled risk ratios (RRs) and 95% confidence intervals (CIs) were calculated for outcome analyses.

## 3. Results

### 3.1. Study Selection and Characteristics

**Figure 1:**
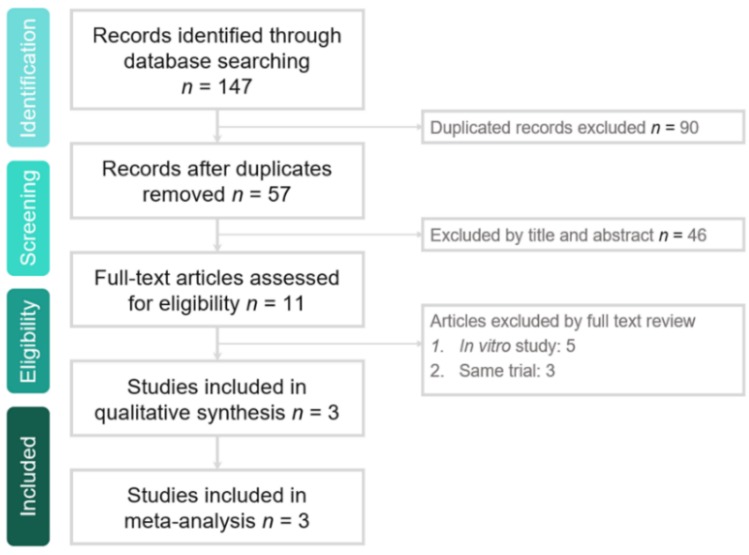
Study selection process flow.

The search program yielded 147 references. After excluding 90 duplications, the remaining 57 abstracts were screened. Among them, we retrieved 11 articles for full-text review. Finally, three studies [11,12,13] fulfilling the inclusion criteria were included in this meta-analysis (Figure 1). All enrolled studies had the same principal investigator. All studies [11,12,13] were randomized, multicenter, and multinational studies designed to compare the clinical efficacy and safety of eravacycline with other comparators for adult patients with cIAI (Table 1). The inclusion criterion of these three studies was that adult patients had to have clinical evidence of cIAI requiring urgent surgical or percutaneous intervention within 48 hours of diagnosis. Two studies [11,13] compared eravacycline with ertapenem, and one [12] compared with meropenem. The test-of-cure evaluation was conducted 25 to 31 calendar days in two studies [11,12] and 10 to 14 days in one study [13] after the first dose of the study drug was administered for the patients with cIAI. The follow-up visit was performed 38 to 50 calendar days in one study [11] and 28 to 42 days in one study [13] after the first dose of study drug was administered. All of the domains in each study were classified as having a low risk of bias (Table 2).

### 3.2. Clinical Efficacy and Microbiologic Response

**Figure 2:**
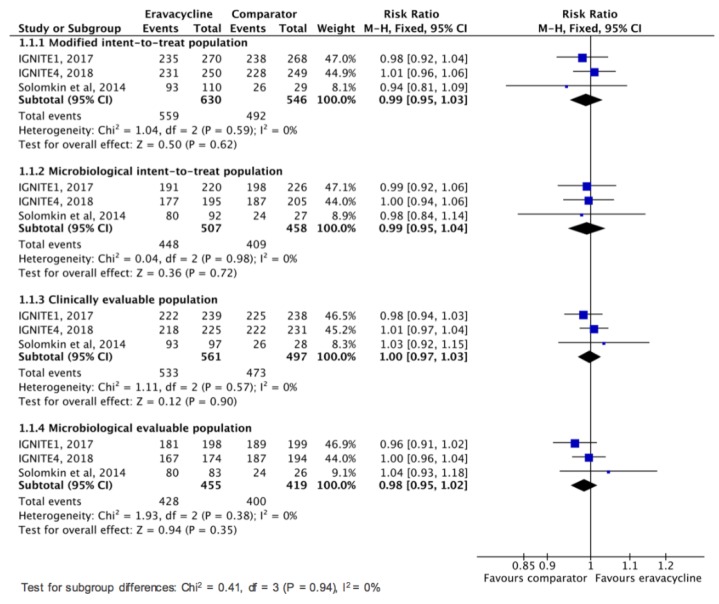
Overall clinical cure rates for eravacycline and comparators in the treatment of complicated intra-abdominal infections.

**Figure 3:**
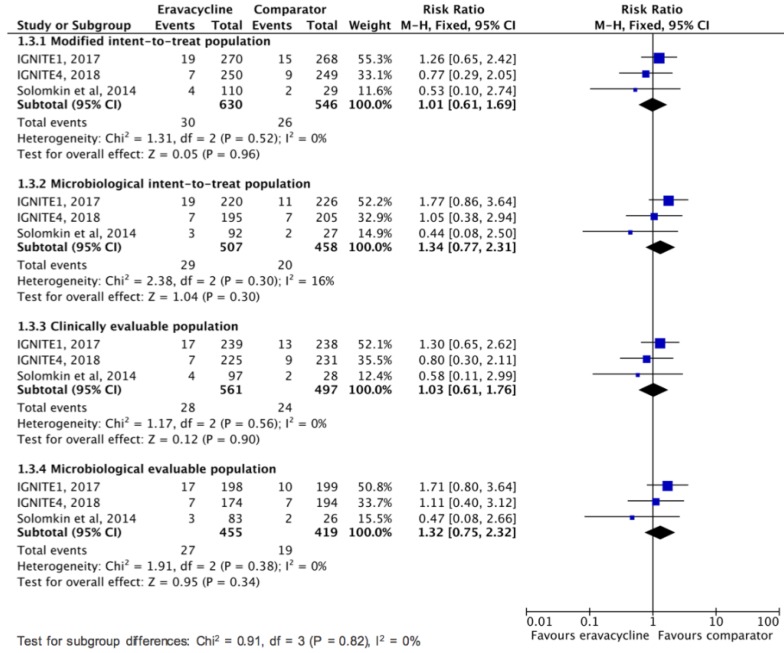
Overall clinical failure rates for eravacycline and comparators in the treatment of complicated intra-abdominal infections.

Overall, eravacycline had a clinical cure rate (88.7%, 559/630) at test-of-cure in modified intent-to-treat population similar to comparators (90.1%, 492/546) in the treatment of cIAIs (risk ratio (RR), 0.99; 95% CI, 0.95–1.03; *I*^2^ = 0%, Figure 2). In the microbiological intent-to-treat, clinically evaluable, and microbiological evaluable populations, no difference was found between eravacycline and comparators in terms of clinical cure rate at test-of-cure (Figure 2). In addition, no significant difference was observed between eravacycline and comparator in terms of clinical failure rate at test-of-cure in modified intent-to-treat population, microbiological intent-to-treat population, clinically evaluable population, and microbiological evaluable population (Figure 3).

Only two studies [12,13] reported the outcome at end-of-treatment, and the pooled analysis showed no significant difference was observed between eravacycline and comparator in terms of clinical cure rate at end-of-treatment in modified intent-to-treat population (RR, 0.99; 95% CI, 0.95–1.03; *I*^2^ = 1%), microbiological intent-to-treat population (RR, 0.98; 95% CI, 0.94–1.03; *I*^2^ = 0%), clinically evaluable population (RR, 0.99; 95% CI, 0.96–1.01; *I*^2^ = 0%), and microbiological evaluable population (RR, 0.98; 95% CI, 0.95–1.01; *I*^2^ = 0%). In addition, these two studies^12,13^ reported the outcome at follow-up, and the pooled analysis showed no significant difference was observed between eravacycline and comparator in terms of clinical cure rate at follow-up in modified intent-to-treat population (RR, 0.99; 95% CI, 0.93–1.04; *I*^2^ = 0%), microbiological intent-to-treat population (RR, 0.98; 95% CI, 0.92–1.06; *I*^2^ = 0%), clinically evaluable population (RR, 1.01; 95% CI, 0.97–1.06; *I*^2^ = 0%), and microbiological evaluable population (RR, 1.02; 95% CI, 0.97–1.07; *I*^2^ = 30%).

### 3.3. Adverse Events

**Figure 4:**
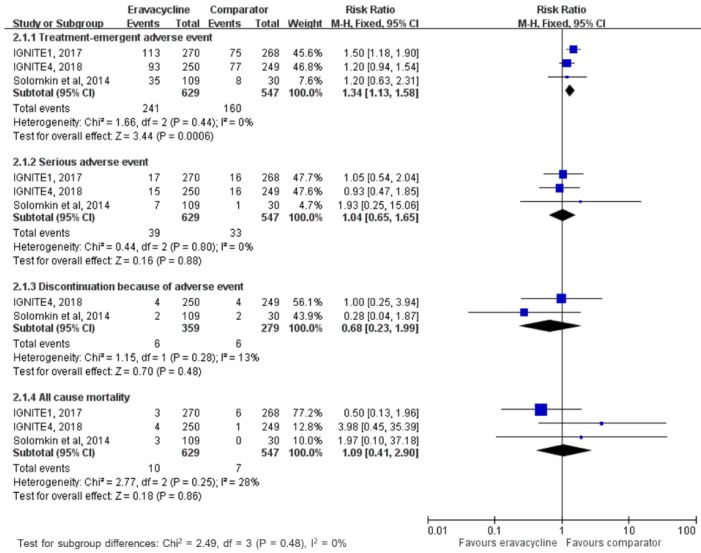
Adverse event risks with eravacycline and comparators in the treatment of complicated intra-abdominal infections.

In the pooled analysis of three studies reporting adverse events, we found that eravacycline was associated with a higher risk of treatment-emergent adverse events than comparators (Figure 4). However, no significant differences were found between eravacycline and comparators for the risk of serious adverse events, discontinuation of study drug because of adverse event, and all-cause mortality (Figure 4). The most common adverse event among the eravacycline group was nausea (6.5%, 41/629) and vomiting (3.8%, 24/629). Although the risks of nausea and vomiting in the eravacycline group were higher than those in the comparator group, these differences did not reach statistical significance (for nausea, RR, 4.79; 95% CI, 0.84–27.14;7 *I*^2^ = 70%, for vomiting, RR, 1.46; 95% CI, 0.76–2.81;7 *I*^2^ = 0%).

## 4. Discussion

This first meta-analysis based on three RCTs [11,12,13] determined that the clinical efficacy of eravacycline is similar to that of other comparators in the treatment of adult patients with cIAIs. This significant finding is supported by the following analysis. First, the overall pooled clinical cure rate at test-of-cure of eravacycline in treating cIAIs was comparable to carbapenems in modified intent-to-treat, microbiological intent-to-treat, clinically evaluable, and microbiological evaluable populations. Second, pooled clinical failure rate at test-of-cure of eravacycline was as low as comparators in modified intent-to-treat, microbiological intent-to-treat, clinically evaluable, and microbiological evaluable populations. Third, this similarity in terms of clinical efficacy between eravacycline and comparators did not change with the timing of the outcome measure at end-of-treatment and follow-up. In summary, all of these findings indicated that eravacycline can be an effective therapeutic option in the treatment of adult patients with cIAIs.

The effectiveness of ceftaroline in the treatment of cIAIs in adult patients can be supported by in vitro studies [7,9,10,15,16]. In the surveillance of 2213 Gram-negative and 2423 Gram-positive pathogens in 13 Canadian hospitals, the minimum inhibitory concentration_90_ (MIC_90)_ ranged from 0.5 to 2μg/mL for 9 species of *Enterobacteriaceae* tested (*n* = 2067) and extended-spectrum β-lactamase producing *E. coli* (*n* = 141) and *K. pneumoniae* (*n* = 21) did not affect the potency of eravacycline in this study [10]. In another survey of more than 4000 Gram-negative pathogens in New York hospitals [7], eravacycline demonstrated great in vitro activity against *Enterobacteriaceae*—*E. coli*, *K. pneumoniae*, *Enterobacter aerogenes*, and *Enterobacter cloacae* with minimum inhibitory concentration_50_ (MIC_50_)/MIC_90_ of 0.12/0.5 μg/mL, 0.25/1 μg/mL, 0.25/1 μg/mL, and 0.5/1 μg/mL, respectively. Moreover, the potent activity was retained against multidrug-resistant (MDR) isolates, including carbapenem nonsusceptible strains [7,9]. In addition to aerobic bacteria, anerobic bacteria play important roles in the cIAIs. Eravacycline showed good in vitro activity against *Bacteroides* spp., *Parabacteroides* spp., and *Clostridioides difficile* (formerly *Clostridium difficile*) and eravacycline remained potent against the strains with tetracycline-specific resistance determinants and MDR anaerobic pathogens [15,16]. Overall, the potent in vitro activity of eravacycline against commonly encountered pathogens causing cIAI largely explains the great in vivo clinical response in this meta-analysis.

In addition to clinical efficacy of eravacycline for the treatment of cIAIs, we should consider the risk of adverse event while prescribing eravacycline. Nausea and vomiting were the most common adverse events, and the overall incidence of these adverse events were higher than those of comparators. Moreover, the pooled risk of treatment-emergent adverse events was higher in the eravacycline group than in the control group. These findings are consistent with previous pooled analysis of IGNITE1 and IGNITE4, in which eravacycline recipients had higher incidence of nausea (6.5 vs. 0.6%) and vomiting (3.7 vs. 2.5%) [17]. In contrast, the incidence of serious adverse events, discontinuation of study drug because of adverse event, and all-cause mortality was similar between eravacycline and comparators. Therefore, the findings of this meta-analysis suggest that although eravacycline is associated with higher risk of mild adverse events than comparator, overall, eravacycline remains as safe as other comparators in the treatment of cIAI among adult patients.

This study has several limitations. First, only three RCTs were considered in this meta-analysis. Second, the usefulness of eravacycline in treating cIAIs was not assessed according to the disease severity. Third, we did not evaluate the correlation between in vitro activity and in vivo response of eravacycline against each specific pathogen, particularly antibiotic-resistant organisms, in this study.

## 5. Conclusions

In conclusion, eravacycline is as good as comparators in terms of efficacy and tolerance in the treatment of cIAI in adult patients.

## Figures and Tables

**Table 1:** Characteristics of included studies.

Study, Published Year	Study Design	Study Site	Study Period	No. of Patients (ITT population)		Dose Regimen	
				Eravacycline	Comparator	Eravacycline	Comparator
Solomkin et al, 2014	Randomized, double-blind trial	19 sites in 6 countries	2011–2012	56 (1.5 mg/kg), 57 (1.0 mg/kg)	30	1.5 mg/kg or 1.0 mg/kg q24 h	Ertapenem 1 g q24 h
IGNITE1, 2017	Randomized, double-blind trial	66 sites in 11 countries	2013–2014	270	271	1.0 mg/kg q12 h	Ertapenem 1 g q24 h
IGNITE4, 2018	Randomized, double-blind trial	65 sites in 11 countries	2016–2017	250	250	1.0 mg/kg q12 h	Meropenem 1 g q8 h

ITT, intention to treat; q, every; h, hour; mg, milligram; g, gram.

**Table 2:** Risk of bias per study and domain.

Risk of Bias	Study		
	IGNITE1, 2017	IGNITE4, 2018	Solomkin et al, 2014
Random sequence generation	low	low	low
Allocation concealment	low	low	low
Blinding of participants and personnel	low	low	low
Blinding of outcome assessment	low	low	low
Incomplete outcome data	low	low	low
Selective reporting	low	low	low

## Appendix A. Search Strategy

**PubMed Search Strategy—Last Searched on 26 May 2019**		**Results**
1	Eravacycline [Title/Abstract] OR TP-434 [Title/Abstract] OR Xerava [Title/Abstract]	74
2	abdom* [Title/Abstract]	330,674
3	1 AND 2	
4	Search (abdom* (Title/Abstract)) AND (((Eravacycline (Title/Abstract)) OR TP-434 (Title/Abstract)) OR Xerava (Title/Abstract))	22
**Web of Science Search Strategy—Last Searched on 26 May 2019**		**Results**
1	(Eravacycline) OR (Xerava) OR (TP-434)	71
2	(abdom*)	269,250
3	1 AND 2	
4	#1 AND #2	20
**EBSCO Search Strategy—Last Searched on 26 May 2019**		**Results**
1	AB Eravacycline OR AB Xerava OR AB TP-434	176
2	AB abdom*	495,125
3	1 AND 2	
4	S1 AND S2	40
**Cochrane Library Search Strategy—Last Searched on 26 May 2019**		**Results**
1	(Eravacycline):ti,ab,kw OR (TP-434):ti,ab,kw OR (Xerava):ti,ab,kw	12
2	(abdom*):ti,ab,kw	40,365
3	1 AND 2	
4	#1 AND #2	5
**Ovid Medline Search Strategy—Last Searched on 26 May 2019**		**Results**
1	(Eravacycline or Xerava or TP-434).ab.	82
2	abdom*.ab	373,974
3	1 AND 2	
4	1 and 2	24
**Embase Search Strategy—Last Searched on 26 May 2019**		**Results**
1	eravacycline:ti,ab,kw OR xerava:ti,ab,kw OR ‘tp 434’:ti,ab,kw	87
2	abdom*:ti,ab,kw	508,670
3	1 AND 2	
4	#1 AND #2	27
**ClinicalTrials.gov Search Strategy—Last Searched on May 26, 2019**		**Results**
1	Eravacycline (completed studies)	9
